# The Efficacy of Cognitive Training on Neuropsychological Outcomes in Mild Cognitive Impairment: A Meta-Analysis

**DOI:** 10.3390/brainsci13111510

**Published:** 2023-10-25

**Authors:** Simona Raimo, Maria Cropano, Mariachiara Gaita, Gianpaolo Maggi, Nicola Davide Cavallo, Maria Dolores Roldan-Tapia, Gabriella Santangelo

**Affiliations:** 1 Department of Psychology, ‘Luigi Vanvitelli’ University of Campania, 81100 Caserta, Italy; maria.cropano@studenti.unicampania.it (M.C.); mariachiara.gaita@unicampania.it (M.G.); gianpaolo.maggi@unicampania.it (G.M.); nicoladavide.cavallo@studenti.unicampania.it (N.D.C.); 2 Department of Psychology, University of Almería, 04120 Almería, Spain; mdroldan@ual.es

**Keywords:** mild cognitive impairment, mild neurocognitive disorder, cognitive training, cognitive functions, meta-analysis

## Abstract

Mild cognitive impairment (MCI) or mild neurocognitive disorder is an intermediate stage of cognitive impairment between normal cognitive aging and dementia. Given the absence of effective pharmacological treatments for MCI, increasing numbers of studies are attempting to understand how cognitive training (CT) could benefit MCI. This meta-analysis aims to update and assess the efficacy of CT on specific neuropsychological test performance (global cognitive functioning, short-term verbal memory, long-term verbal memory, generativity, working memory, and visuospatial abilities) in individuals diagnosed with MCI, as compared to MCI control groups. After searching electronic databases for randomized controlled trials, 31 studies were found including 2496 participants. Results showed that CT significantly improved global cognitive functioning, short-term and long-term verbal memory, generativity, working memory, and visuospatial abilities. However, no significant effects were observed for shifting, abstraction ability/concept formation, processing speed, and language. The mode of CT had a moderating effect on abstraction ability/concept formation. The findings provide specific insights into the cognitive functions influenced by CT and guide the development of tailored interventions for MCI. While CT holds promise, further research is needed to address certain cognitive deficits and assess long-term effects on dementia progression.

## 1. Introduction

Mild cognitive impairment (MCI), also known as mild neurocognitive disorder (mild NCD), is an intermediate state between normal cognition and dementia. It is characterized by a slight cognitive decline that does not have a significant impact on independence in daily activities [1]. While a substantial number of people with MCI maintain stability or revert to normal cognition over time (about 16% [2]), over half of them progress to dementia within five years [3]. To mitigate cognitive decline, individuals with MCI may turn to non-pharmacological interventions, such as cognitive interventions. These interventions are broadly categorized into three approaches: cognitive stimulation, cognitive training (CT), and cognitive rehabilitation [4]. More specifically, cognitive stimulation reflects mentally stimulating activities in which the patient participates to improve cognition and social functioning; conversely, CT is usually conceived as individual or group sessions aimed at enhancing specific cognitive functions such as memory or executive exercises through paper and pencil instruments or computerized tools. Finally, cognitive rehabilitation involves tailored interventions designed and implemented to recover cognitive abilities temporarily and partially lost and to address each patient’s key difficulties and goals [4]. Among these, CT has demonstrated the highest efficacy in enhancing cognition and improving psychosocial functioning in both healthy and clinical populations [5,6,7].

Specifically, CT involves structured practice on standardized cognitive tasks designed to enhance specific cognitive functions like memory, attention, or language [8,9]. These tasks can be administered individually or in group settings, using paper-and-pencil or computerized formats, and sometimes can simulate activities of daily life [10]. A fundamental assumption of CT is that practice can either improve, or, at least, maintain, functioning in the targeted cognitive domain, with effects extending beyond the training context [9].

Numerous studies have investigated the efficacy of CT in treating neuropsychological profiles in MCI using both unimodal (solely CT) and multimodal interventions (e.g., CT combined with physical fitness or drug treatment). These studies have reported significant enhancements in cognitive abilities and daily living skills among older individuals with MCI [10,11,12,13]. These interventions may have tapped into pre-existing cognitive reserves [14,15] and facilitated neuroplasticity in various brain regions, including the frontoparietal network and the hippocampus—a critical region for memory support [16,17]. However, the results of CT research have not always been consistent. While some studies have found clear benefits for trained ability, including both near and far effects on untrained abilities, other research has yielded little to no evidence of CT’s benefit. These disparities in findings may be attributed to heterogeneity in the design and methodological rigor of CT studies, limiting the understanding of the mechanisms of CT and its applicability to different populations [18].

Furthermore, previous systematic reviews and meta-analyses examining the efficacy of cognitive interventions in MCI have reported mixed results, e.g., [6,18,19]. These reviews have often failed to differentiate between various forms of interventions and training, the instruments used to measure outcomes, and the inclusion of a control group. To address these limitations found in the existing literature, the current meta-analysis aims to update and assess the efficacy of CT on specific neuropsychological test performances in individuals diagnosed with MCI, as compared to MCI control groups (i.e., individuals engaged in alternative training, like mental leisure activities and usual care) in randomized controlled trials (RCTs). We sought to address the following research questions: (i) which cognitive domains exhibit improvement after the application of CT in MCI?; and (ii) what are the effects of relevant demographic, clinical, and CT characteristics (e.g., duration and frequency of CT, type of CT) on the calculated effect sizes? Identifying the specific cognitive variables that improve following CT in MCI could provide valuable insights for enhancing the management and planning of potential cognitive remediation interventions aimed at preventing cognitive decline and the associated loss of functionality.

## 2. Materials and Methods

### 2.1. Study Registration

**Figure 1:**
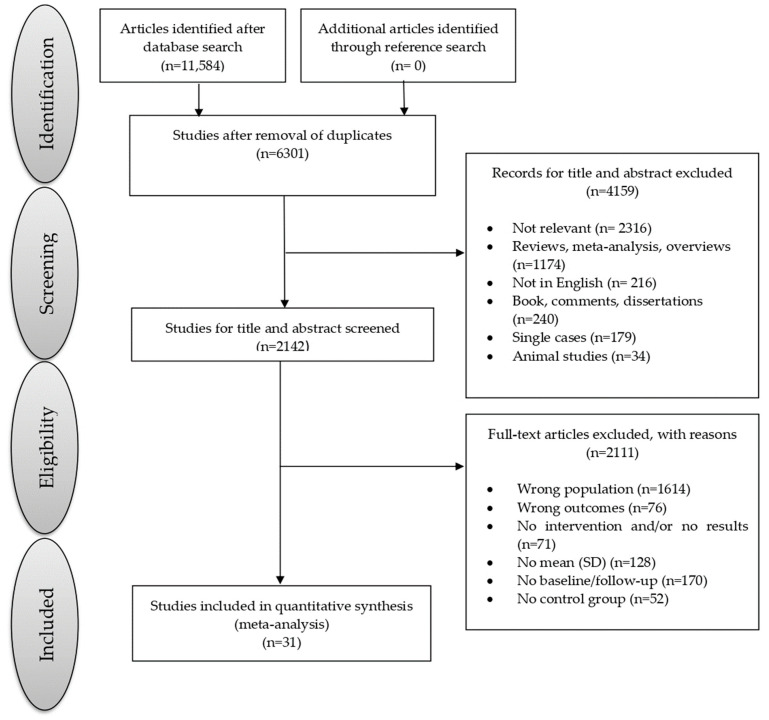
Flowchart of the selection process of primary studies.

The current meta-analysis was preregistered electronically on the PROSPERO International prospective register of systematic reviews under the registration number CRD42023421038. It was conducted in accordance with the PRISMA (Preferred Reporting Items for Systematic Reviews and Meta-Analyses) guidelines [20,21]. The study selection process is visually represented in Figure 1.

### 2.2. Data Sources and Study Selection

Article searches were comprehensively conducted on the PsycInfo (PROQUEST), PubMed, and Scopus databases. The search query utilized the term string: “cognitive training” AND (cognit* OR memory OR attention OR “executive function” OR Language) AND (MCI OR “mild cognitive impairment” OR “mild neurocognitive disorder”) AND (longitudinal OR “follow-up” OR outcomes OR predict*). The final date for the database searches was the end of March 2023. Peer-reviewed English studies that evaluated cognitive status in MCI were included if they met the following criteria: first, the study should compare individuals diagnosed with MCI or mild NCD undergoing CT against individuals diagnosed with MCI or mild NCD engaged in an alternative control training (e.g., active interventions like non-adaptive/non-tailored CT, educational activities on cognitive functions; or passive interventions like mental leisure activities or usual care); second, the study should assess cognitive functioning using a validated neuropsychological battery composed of recognized neuropsychological tests; third, the study should have provided adequate data (e.g., mean and median) to compute effect sizes for cognitive outcomes, both before and after the execution of the CT. The primary studies with the highest number of participants were selected when 2 or more studies provided data obtained from the same database [22].

### 2.3. Screening and Data Extraction

Article screening, data extraction, and quality evaluation were independently conducted by two investigators (SR and MC). The extracted information included: (i) publication characteristics; (ii) sample characteristics; (iii) CT characteristic; and (iv) means and standard deviations of raw scores or age- and education-adjusted scores, as reported in primary studies, for neuropsychological tests used to assess cognitive domains. To assess the overall external and internal validity and analysis of studies included in the meta-analysis, we calculated a quality score according to a modified version of the Newcastle–Ottawa Quality Assessment Scale [23].

### 2.4. Outcomes

The outcomes assessed in this meta-analysis included: global cognitive functioning, memory, executive functions, and their subdomains (i.e., shifting, inhibition, generativity, and working memory), processing speed/attention, visuospatial abilities, and language. We categorized neuropsychological tasks into the aforementioned cognitive domains based on the indication provided by primary studies. To avoid higher levels of inter-study heterogeneity, we selected for each cognitive domain the most frequently utilized neuropsychological task from the primary studies, ensuring it was performed by the largest number of participants, serving as the outcome for the meta-analysis. A list of the neuropsychological tasks corresponding to each cognitive domain can be found in Supplementary Material Table S1.

### 2.5. Statistical Analysis

Statistical analyses were conducted utilizing ProMeta 3.0 software (Intenovi 2015, Cesena, Italy). To achieve the primary objectives of the meta-analysis, we calculated effect sizes (ES) from the data reported in the primary studies using the Hedges unbiased approach [24]. Hedges’g-values were interpreted according to the following conventions: values < 0.20 indicated small effects, values around 0.50 suggested moderate effects, and values near 0.80 indicated large effects. Specifically, we examined the following comparisons of interest: (i) individuals with MCI involved in cognitive training (+CT) compared to those engaged in control training (−CT). Positive Hedges’ g-values indicated that individuals with MCI + CT scored higher than those with MCI − CT. Heterogeneity among the studies was evaluated using the Q and I^2^ statistics index [25]. We explored the impact of demographic and clinical characteristics (such as age, gender, years of education, and type of MCI), type of training (computerized or traditional), mode of training (unimodal or multimodal intervention), and duration of CT (intervention duration in weeks, session duration in minutes, and sessions per week), type of control group (i.e., passive or active), through several meta-regressions. The presence of publication bias was assessed using a scatter plot of estimated ES from individual studies against a measure of study precision (e.g., their standard errors [26]). To ensure a more robust funnel plot analysis, we utilized Egger’s regression method [27]. Furthermore, we employed the trim-and-fill procedure [28] to assess the potential impact of data censoring on the outcomes of the meta-analysis. According to this method, studies that introduce asymmetry in funnel plots are identified and adjusted, allowing for the overall effect estimate derived from the remaining studies to be minimally influenced by publication bias. A *p*-value of <0.05 was set as the cut-off for significance in all analyses.

## 3. Results

### 3.1. Study Characteristics

A total of 11,584 studies were identified through the search process. After assessing the title and abstract, the full texts of these 31 studies were obtained, enrolling a cumulative total of 2496 individuals with MCI (mean age = 73.86 years, SD = 6.51 years; mean education = 10.92 years, SD = 3.18 years, 32.45% male). Specifically, of the 2496 individuals with MCI, 1310 participated in CT (mean age = 73.76 years, SD = 6.37 years; mean education = 11.01 years, SD = 3.04 years; 32.36% male; mean intervention duration = 12.11 weeks; mean session duration = 73.39 min; mean session for week = 2.28 times), while 1186 were included in a control training group (mean age = 73.97 years, SD = 6.66 years, mean education = 10.83 years, SD = 3.32 years, 32.54% male; mean intervention duration = 13.53 week; mean session duration = 60.41 min; mean session for week = 2.58 times). Details regarding the demographic and clinical characteristics of participants, as well as the type of training, are presented in Table 1. The table also provides results from the quality assessment based on the Newcastle–Ottawa quality assessment scale; the quality scores of the included studies ranged from 6 to 10, indicating relatively high quality and, consequently, yielding reliable findings.

### 3.2. Meta-Analytic Results

#### 3.2.1. Global Cognitive Functioning

**Figure 2:**
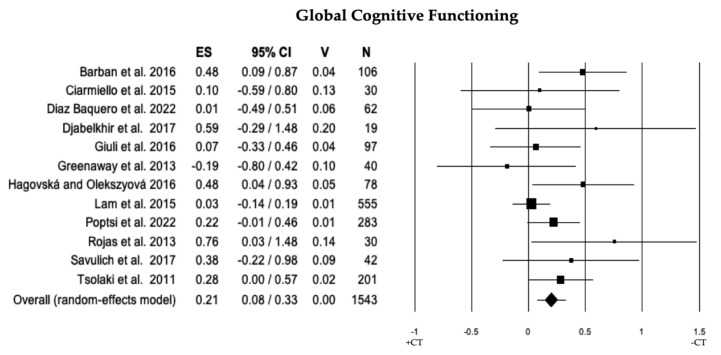
Forest plot for global cognitive functioning domain illustrating comparison between individuals with MCI involved in cognitive training (+CT) versus those involved in control training (−CT), displaying effect size (Hedges’ g) calculated using a random-effects model. ES = effect size; CI = confidence intervals; V = variance; N = total number of participants [30,33,35,36,40,41,42,46,52,54,56,58].

In 12 studies, a comparison of global cognitive functioning between individuals with MCI + CT and those with MCI − CT was conducted. Individuals with MCI + CT demonstrated significantly higher scores than those with MCI − CT (ES = 0.21). The heterogeneity was low (I^2^ = 20.73), and no publication bias was observed (*p* = 0.139). However, the trim and fill analysis removed two studies revealing a difference between overall observed ES (0.21) and overall estimated ES (0.18; see Figure 2 and Table 2).

#### 3.2.2. Short-Term Verbal Memory

**Figure 3:**
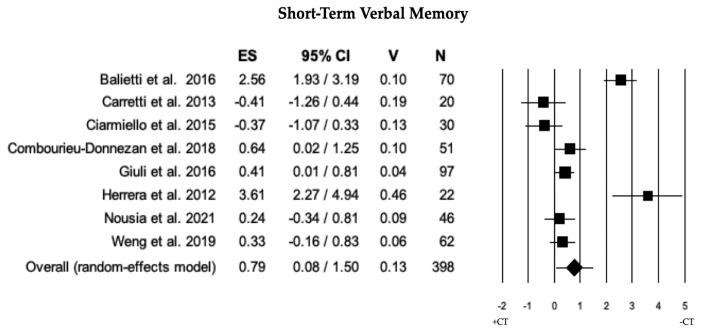
Forest plot for short-term verbal memory (memory domain) illustrating comparison between individuals with MCI involved in cognitive training (+CT) versus those involved in control training (−CT), displaying effect size (Hedges’ g) calculated using a random-effects model. ES = effect size; CI = confidence intervals; V = variance; N = total number of participants [29,31,33,34,40,43,50,59].

Across 8 studies, a comparison of short-term verbal memory between individuals with MCI + CT and those with MCI − CT was performed. Individuals with MCI + CT displayed significantly higher scores than those with MCI − CT (ES = 0.79). The heterogeneity was high (I^2^ = 90.42), and there was neither publication bias (*p* = 0.417) nor any trimmed studies (see Table 2 and Figure 3).

#### 3.2.3. Long-Term Verbal Memory

**Figure 4:**
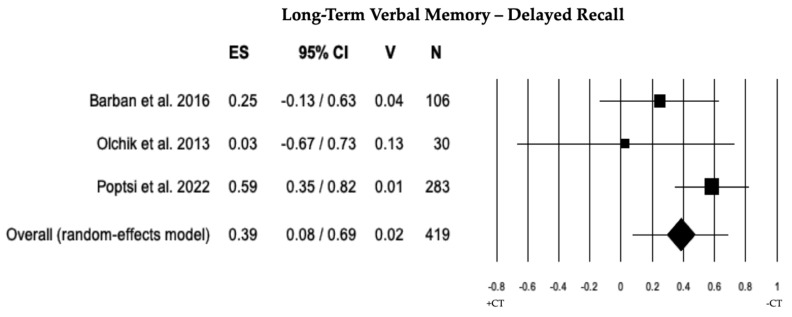
Forest plot for long-term verbal memory (memory domain) illustrating comparison between individuals with MCI involved in cognitive training (+CT) versus those involved in control training (−CT), displaying effect size (Hedges’ g) calculated using a random-effects model. ES = effect size; CI = confidence intervals; V = variance; N = total number of participants [30,51,52].

In 4 studies, a comparison of long-term verbal memory between individuals with MCI + CT and those with MCI − CT was conducted. Individuals with MCI + CT exhibited significantly higher scores than those with MCI − CT (ES = 0.31). The heterogeneity across studies was moderate (I^2^ = 46.86), and there was evidence of publication bias (*p* = 0.042), although no trimmed studies were identified. A sensitivity analysis recommended the exclusion of one study [33], revealing a significant ES (0.39). The heterogeneity remained moderate (I^2^ = 45.80), and no publication bias (*p* = 0.231) or trimmed studies were observed (see Table 2 and Figure 4).

#### 3.2.4. Shifting

**Figure 5:**
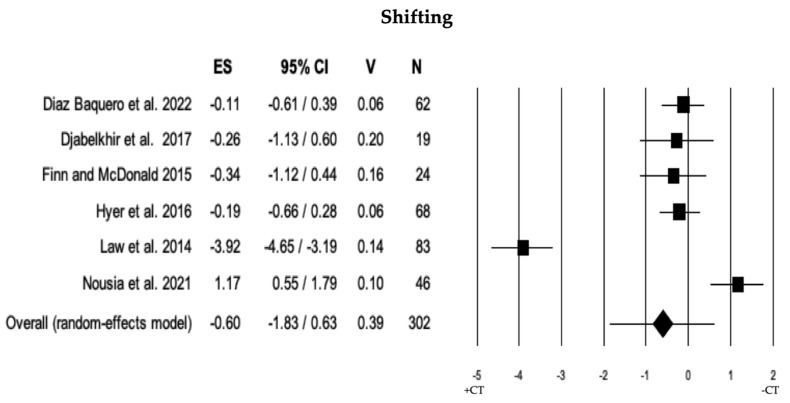
Forest plot for shifting ability (executive domain) illustrating comparison between individuals with MCI involved in cognitive training (+CT) versus those involved in control training (−CT), displaying effect size (Hedges’ g) calculated using a random-effects model. ES = effect size; CI = confidence intervals; V = variance; N = total number of participants [35,36,39,45,47,50].

The comparison of shifting ability was conducted in 6 studies between individuals with MCI + CT and those with MCI − CT. The results indicated that there was no significant difference between individuals with MCI + CT and those with MCI − CT (ES = −0.60). The heterogeneity was high (I^2^ = 95.67), and no publication bias was detected (*p* = 0.529). However, during the trim and fill analysis, two studies were removed, revealing a discrepancy between the overall observed ES (−0.60) and the overall estimated ES (−1.18; see Table 2 and Figure 5).

#### 3.2.5. Abstraction Ability/Concept Formation

**Figure 6:**
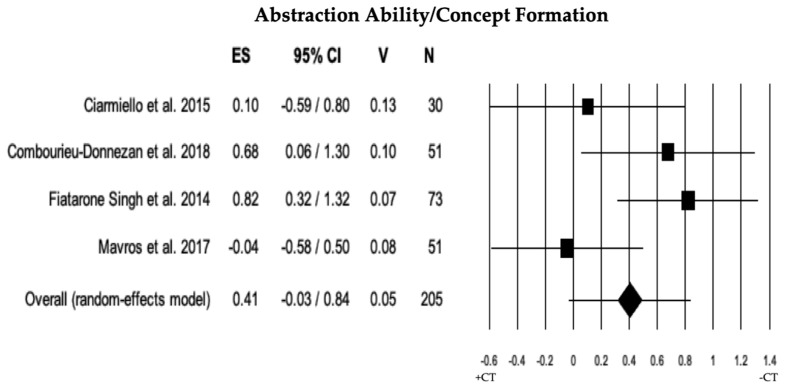
Forest plot for abstraction ability/concept formation (executive domain) illustrating comparison between individuals with MCI involved in cognitive training (+CT) versus those involved in control training (−CT), displaying effect size (Hedges’ g) calculated using a random-effects model. ES = effect size; CI = confidence intervals; V = variance; N = total number of participants [33,34,38,49].

In 4 studies, the comparison of abstraction ability/concept formation between individuals with MCI + CT and those with MCI − CT was conducted. The results indicated that there was a trend toward a significant difference between individuals with MCI + CT and those with MCI − CT (ES = 0.41); however, without reaching statistical significance based on 4 eligible studies (*p* = 0.069). The heterogeneity was moderate (I^2^ = 55.32), and there was no publication bias (*p* = 0.652) or any trimmed studies (see Table 2 and Figure 6).

#### 3.2.6. Generativity

**Figure 7:**
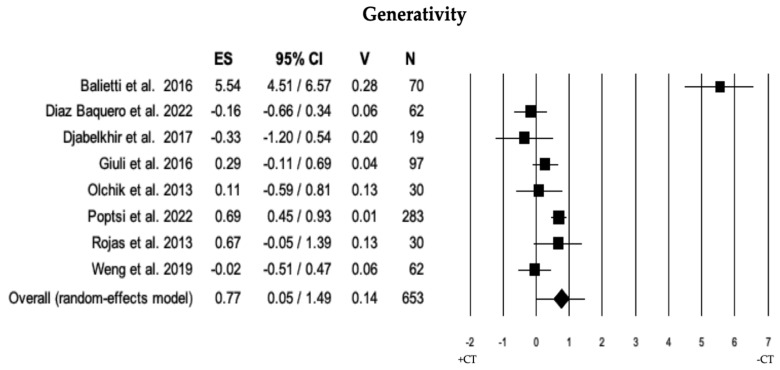
Forest plot for generativity (executive domain) illustrating comparison between individuals with MCI involved in cognitive training (+CT) versus those involved in control training (−CT), displaying effect size (Hedges’ g) calculated using a random-effects model. ES = effect size; CI = confidence intervals; V = variance; N = total number of participants [29,35,36,40,51,52,54,59].

Across 8 studies, the comparison of generativity between individuals with MCI + CT and those with MCI − CT was performed. Individuals with MCI + CT scored significantly higher than those with MCI − CT (ES = 0.77). The heterogeneity was high (I^2^ = 93.73), and neither publication bias (*p* = 0.635) nor trimmed studies were observed (see Table 2 and Figure 7).

#### 3.2.7. Working Memory

**Figure 8:**
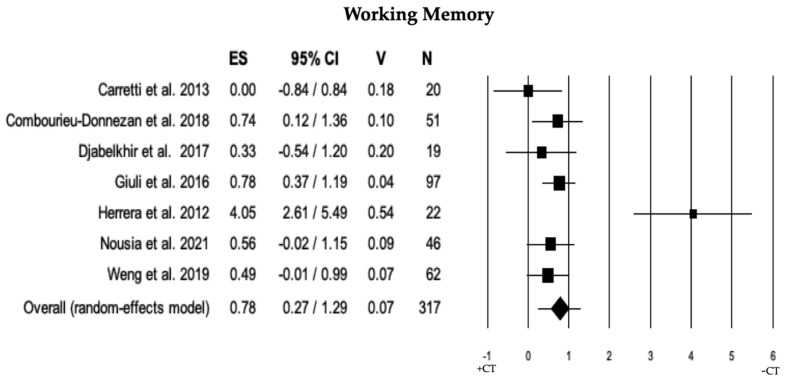
Forest plot for working memory (executive domain) illustrating comparison between individuals with MCI involved in cognitive training (+CT) versus those involved in control training (−CT), displaying effect size (Hedges’ g) calculated using a random-effects model. ES = effect size; CI = confidence intervals; V = variance; N = total number of participants [31,34,36,40,43,50,59].

In 7 studies, the comparison of working memory between individuals with MCI + CT and those with MCI − CT was performed. Individuals with MCI + CT scored significantly higher than those with MCI − CT (ES = 0.78). The heterogeneity was high (I^2^  =  76.06), and there was no publication bias (*p*  =  0.325) or trimmed studies (see Table 2 and Figure 8).

#### 3.2.8. Processing Speed

**Figure 9:**
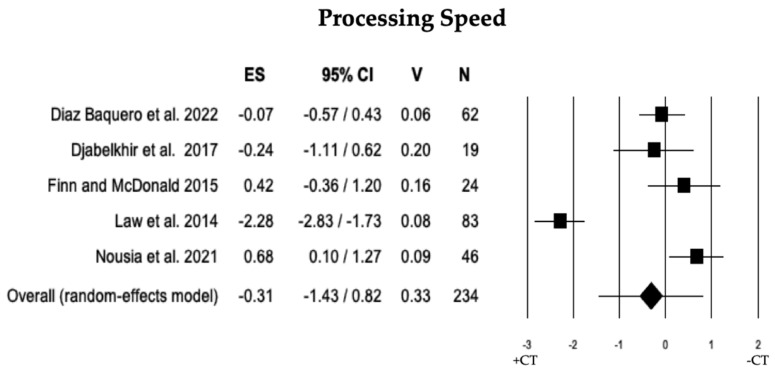
Forest plot for processing speed (executive domain) illustrating comparison between individuals with MCI involved in cognitive training (+CT) versus those involved in control training (−CT), displaying effect size (Hedges’ g) calculated using a random-effects model. ES = effect size; CI = confidence intervals; V = variance; N = total number of participants [35,36,39,47,50].

The comparison of processing speed was conducted in 5 studies between individuals with MCI + CT and those with MCI − CT. The results indicated that there was no significant difference between individuals with MCI + CT and those with MCI − CT (ES = −0.31). The heterogeneity was high (I^2^ = 93.75), and no publication bias was detected (*p* = 0.697). However, during the trim and fill analysis, one study was removed, revealing a discrepancy between the overall observed ES (−0.31) and the overall estimated ES (−0.56; see Table 2 and Figure 9).

#### 3.2.9. Visuospatial and Constructional Ability

**Figure 10:**
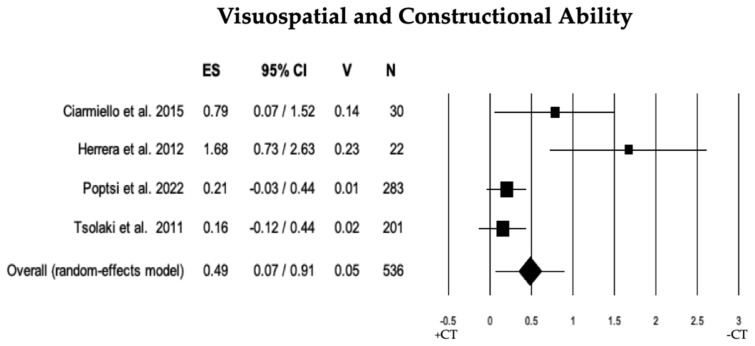
Forest plot for visuospatial and constructional ability illustrating comparison between individuals with MCI involved in cognitive training (+CT) versus those involved in control training (−CT), displaying effect size (Hedges’ g) calculated using a random-effects model. ES = effect size; CI = confidence intervals; V = variance; N = total number of participants [33,43,52,58].

The comparison of visuospatial and constructional abilities between individuals with MCI + CT and those with MCI − CT was performed across 4 studies. Individuals with MCI + CT scored significantly higher than those with MCI − CT (ES = 0.49). However, there was notable heterogeneity (I^2^ = 73.67) among the studies. Despite this, no publication bias was detected (*p* = 0.054). A trim and fill analysis was performed, which resulted in the exclusion of one study. This adjustment revealed a disparity between the overall observed ES (0.49) and the overall estimated ES (0.31; see Table 2 and Figure 10).

#### 3.2.10. Language

**Figure 11:**
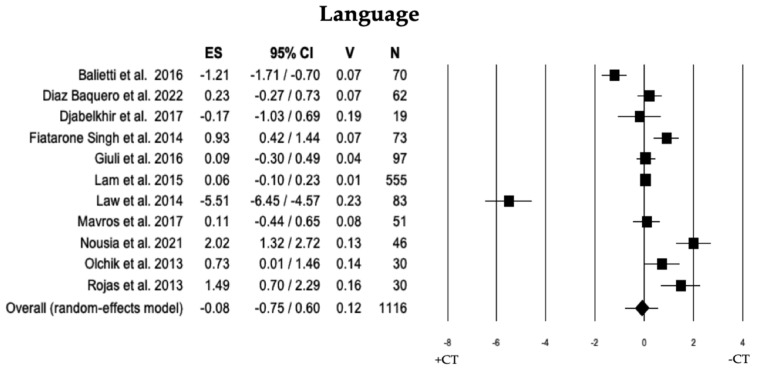
Forest plot for language domain illustrating comparison between individuals with MCI involved in cognitive training (+CT) versus those involved in control training (−CT), displaying effect size (Hedges’ g) calculated using a random-effects model. ES = effect size; CI = confidence intervals; V = variance; N = total number of participants [29,35,36,38,40,46,47,49,50,51,54].

Across 11 studies, a comparison was performed on language abilities between individuals with MCI + CT and those with MCI − CT. Individuals with MCI + CT and those with MCI − CT did not exhibit a significant difference (ES = −0.08). The studies showed substantial heterogeneity (I^2^ = 95.39), and no publication bias was identified (*p* = 0.810). However, following a trim and fill analysis, 4 studies were excluded, revealing a difference between overall observed ES (−0.08) and overall estimated ES (−0.85; see Table 2 and Figure 11).

### 3.3. Moderator Analysis

We found that the mode of CT moderated performance on task evaluating abstraction ability/concept formation. Specifically, the multimodal interventions of CT were associated with a higher performance (Q(1) = 6.49, *p* = 0.011). Neither the effects of other demographic, clinical, and CT variables (i.e., age, sex, years of education, type of MCI, type of control group, intervention duration in weeks, session duration in minutes, and session for a week) were statistically significant.

## 4. Discussion

The current meta-analysis provides new insights into the effectiveness of CT in MCI through a systematic exploration of studies that compared individuals with MCI + CT to those with MCI − CT on a comprehensive neuropsychological battery to identify the cognitive functions that improve after training. Its findings contribute to the existing literature in several significant ways. First, the results provide a detailed and precise comprehension of the cognitive functions influenced by CT in individuals with MCI. This specificity is crucial for tailoring interventions that target distinct cognitive deficits, potentially improving the overall quality of life for affected individuals [60]. Notably, moderate to high positive ES with statistical significance was found in verbal memory, generativity, working memory, and visuospatial abilities. However, no significant effects were found for abstraction ability/concept formation, processing speed, and language. The improvements on memory and visuospatial abilities domains are unsurprising given their central focus in most interventions and promising given this is the primary complaint in most cases of MCI [61]. Indeed, memory and visuospatial impairments are common and often debilitating in MCI, and the findings suggest that CT can effectively address memory-related difficulties [62]. Additionally, the improvement in working memory and generativity would suggest that CT would have positive effects also on higher-order cognitive abilities mediated by the frontal brain networks, and necessary to maintain independence in activities of daily living (ADL) and delaying dementia development.

These results partly differ from previous meta-analyses that found small to moderate effects on global cognitive functioning [63,64], attention, working memory, learning, and memory [63] after CT in people with MCI. These discrepancies could be due to the different inclusion criteria used in these studies, in particular, they considered only computerized CT and selected for each cognitive domain different types of neuropsychological tests while in the present study, we included computerized or traditional CT and identified the most frequently utilized neuropsychological task from the primary studies for each cognitive domain to avoid higher levels of inter-study heterogeneity.

On the other hand, the lack of effects on shifting and abstraction ability/concept formation might indicate that CT approaches employed in the meta-analysis included studies might not have effectively targeted these cognitive domains, with a lack of far transfer. This issue encourages clinicians to implement and refine CT specifically targeting these cognitive domains to have significant benefits [65,66]. In particular, we found that the type of intervention (unimodal or multimodal) moderated performance on task evaluating abstraction ability/concept formation with multimodal interventions (CT combined with physical exercise) resulted in being more effective in enhancing these abilities compared to interventions involving CT alone. These findings are in line with previous randomized control studies demonstrating that CT immediately preceded by aerobic exercise improved multiple cognitive processes due to the benefic effect of cortisol on learning and memory produced by moderate-intensity physical exercise [67,68]. Otherwise, no significant results were also found for processing speed and language abilities between individuals with MCI + CT and those with MCI − CT. However, the high heterogeneity in these results would suggest that the effectiveness of CT might vary across different studies and interventions. Moreover, the moderator effect of CT mode on abstraction ability/concept formation (i.e., the combined mode of CT was associated with higher performance in this cognitive domain) would encourage us to consider how different CT designs may play a role in their efficacy in certain cognitive domains within clinical setting [69,70].

Thus, the present meta-analyses updated the previous meta-analytic results [18,71], by reducing heterogeneity through the selection of widely used cognitive tests for each domain and considering executive functioning in its different subcomponents (i.e., generativity, abstraction ability/concept formation, working memory, and shifting). It also expands analysis to previously unexamined cognitive domains due to the limited number of primary studies. Nevertheless, this study is not without limitations. First, separate meta-analyses between amnestic and non-amnestic forms of MCI were not conducted. This might lead to significantly different effects among participants and make it difficult to evaluate the effectiveness of the CT and the generalizability of the current results. However, the selection of primary studies with established clear diagnostic criteria for MCI, and implementing RCTs reduced significantly the number of selected studies to perform a deeper analysis. Second, the lack of long-term follow-up made it unclear whether observed post-intervention benefits contributed to delaying or preventing progression from MCI to dementia. Moreover, we should underline that we did not explore the duration necessary to obtain long-term maintenance of benefits. However, although it has been proved that CT performed every week for one year approximately generated improvements in cognitive functions observed also 4 years after the end of the training, the duration seems to have a null on outcome measures [67]. Finally, we suggested taking the results of our meta-analyses with a limited number of studies (i.e., 2–4), with caution as heterogeneity cannot be reliably estimated and can happen a significant statistically moderate or high effect when combining few statistically significant studies with effects pointing into the same direction [72]. These limitations may be potentially overcome by more RCTs examining long-term cognitive outcomes to assess the transfer of CT to daily life and provide more insight into its impact on dementia progression.

## 5. Conclusions

In conclusion, the results from the meta-analysis provide valuable new insight into the efficacy of CT in MCI. By thoroughly examining cognitive domains and identifying specific functions that improve with training, this study offers guidance for the development of targeted and effective interventions to support individuals with MCI. The findings underscore the potential benefits of CT in enhancing cognitive functioning and quality of life within this population. Nevertheless, the study also highlights areas where further research and refinement of CT approaches are needed to effectively address certain cognitive deficits.

## Figures and Tables

**Table 1:** Demographic and cognitive training characteristics of primary studies included in the meta-analysis.

	Demographic Characteristic				Type of Training	Cognitive Training Characteristic			Neuropsychological Assessment			Quality Score
	N	Age	Education	n M		Intervention Duration (Weeks)	Session Duration (Minutes)	Session for Week	Cognitive Test	Baseline Means (SD)	Follow-Up Means (SD)	
Balietti et al. [29] (MCI)	37	N.R.	N.R.	N.R.	Cognitive Training	10	60	1	Digit span forward	4.36 (0.15)	4.65 (0.16)	7
									Corsi supraspan	4.84 (0.16)	5.17 (0.15)	
									Attentive matrices	39.65 (1.66)	43.39 (1.60)	
									Semantic verbal fluency	1.84 (0.23)	2.13 (0.28)	
									Phonological verbal fluency	28.46 (1.31)	31.27 (1.60)	
									Prose memory	6.83 (0.68)	8.8 (0.66)	
									Word-pairing test	8.65 (0.72)	8.98 (0.82)	
	33	N.R.	N.R.	N.R.	Active Control Training	N.R.	N.R.	N.R.	Digit span forward	4.56 (0.13)	4.47 (0.13)	
									Corsi supraspan	5.12 (0.12)	5.01 (0.13)	
									Attentive matrices	41.32 (1.57)	40.04 (1.43)	
									Semantic verbal fluency	2.42 (0.24)	2.40 (0.22)	
									Phonological verbal fluency	24.83 (1.36)	24.01 (1.03)	
									Prose memory	7.55 (0.76)	6.71 (0.76)	
									Word-pairing test	6.91 (0.53)	7.04 (0.50)	
Barban et al. [30] (MCI)	46	74.4 (5.7)	9 (4.3)	25	Computer- based Cognitive Training	12	60	2	MMSE	27.3 (2.1)	27.9 (1.9)	8
									RAVLT delayed recall	4.1 (3.2)	5 (3.6)	
	60	72.9 (6)	11 (4.7)	31	Passive Control Training	-	-	-	MMSE	28.1 (1.4)	27.7 (2.2)	
									RAVLT delayed recall	5 (3.2)	5 (3.6)	
Carretti et al. [31] (a-MCI)	10	71.8 (2.20)	6.50 (2.83)	6	Cognitive Training	5	90	1	Dot matrix	5.60 (2.45)	7.90 (0.99)	8
									Digit span forward	5.40 (1.77)	5.2 (1.68)	
									Digit span backward	4 (1.63)	4.70 (1.05)	
									List recall	3 (1.15)	4.40 (0.96)	
									Pattern comparison task	153.5 (42.4)	63.6 (55.97)	
									Cattel test	13.70 (4.76)	17.40 (4.47)	
	10	70.6 (2.63)	7.20 (3.29)	4	Active Control Training	5	90	1	Dot matrix	6.70 (2.45)	7.30 (2.05)	
									Digit span forward	5.10 (1.44)	5.50 (1.08)	
									Digit span backward	3.60 (0.84)	4.30 (1.16)	
									List recall	3.30 (1.76)	3.60 (1.77)	
									Pattern comparison task	142.70 (43.60)	141.60 (27.75)	
									Cattel test	14.30 (4.21)	13.90 (3.69)	
Chen et al. [32] (a-MCI)	46	75.13 (7.56)	15.98 (2.29)	27	Vision-based speed of processing training	6	60	4	UFOV	5.87 (0.53)	5.52 (0.49)	6
	28	73.68 (6.92)	16.68 (2.87)	13	Active Control Training	6	60	4	UFOV	5.89 (0.50)	5.76 (0.48)	
Ciarmiello et al. [33] (MCI)	15	71.22 (7.66)	9.30 (3.02)	5	Computer- based Cognitive Training	16	45	2	MMSE	27.87 (1.82)	28.26 (1.54)	8
									Matrix reasoning test	29.34 (4.56)	30.61 (3.89)	
									Digit span forward	5.21 (0.81)	5.39 (1.02)	
									Word span	4.21 (0.63)	4.13 (0.48)	
									Corsi span	4.54 (0.53)	4.72 (0.68)	
									Prose memory	10.44 (2.21)	11.57 (2.04)	
									Rey figure copy	31.56 (2.81)	32.05 (3.50)	
									Rey figure delayed recall	12.64 (2.95)	16.26 (7.55)	
									RAVLT immediate recall	32.15 (7.66)	33.91 (7.82)	
									RAVLT delayed recall	6.09 (2.91)	6.32 (2.91)	
	15	71.95 (7.13)	7.83 (2.63)	7	Passive Control Training	-	-	-	MMSE	27.83 (1.86)	28.04 (1.85)	
									Matrix reasoning test	27.07 (6.64)	27.83 (5.53)	
									Digit span forward	5.14 (0.66)	5.63 (0.55)	
									Word span	4.19 (0.41)	4.13 (0.48)	
									Corsi span	4.36 (0.93)	4.63 (0.98)	
									Prose memory	9.92 (3.11)	9.97 (3.09)	
									Rey figure copy	31.88 (4.06)	29.40 (3.79)	
									Rey figure delayed recall	15.49 (6.48)	14.65 (5.60)	
									RAVLT immediate recall	33.32 (8.17)	33.69 (9.38)	
									RAVLT delayed recall	6.85 (2.95)	7.27 (3.26)	
Combourieu- Donnezan et al. [34] (MCI)	21	75.2 (1.3)	5.9 (0.31)	N.R.	Physical and Cognitive Training	12	60	2	Matrix reasoning test	12.38 (6.48)	16.42 (7.33)	9
									Stroop	26.52 (6.8)	29.05 (7.19)	
									Digit span forward	5.48 (0.88)	6.15 (1.06)	
									Digit span backward	4.10 (1.18)	4.95 (1.12)	
	16	76.3 (1.5)	5.5 (0.36)	N.R.	Cognitive Training	12	60	2	Matrix reasoning test	11.35 (6.84)	15.18 (6.84)	
									Stroop	27.19 (8.82)	25.5(8.37)	
									Digit span forward	5.18 (0.91)	6.18 (1.11)	
									Digit span backward	4.06 (0.68)	4.63 (1.09)	
	14	79.2 (4)	5.8 (0.4)	N.R.	Passive Control Training	-	-	-	Matrix reasoning test	9.85 (6.21)	9.5 (4.95)	
									Stroop	24.71 (10.16)	26.42 (6.53)	
									Digit span forward	5.21 (1.12)	5.36 (0.84)	
									Digit span backward	3.86 (0.86)	3.79 (0.97)	
Diaz Baquero et al. [35] (MCI)	36	73.64 (6.56)	9.35 (2.60)	13	Computer- based Cognitive Training	16	30	2/3	MMSE	24.28 (2.54)	24.97 (2.91)	9
									ADAS-Cog	14.00 (4.99)	14.43 (6.48)	
									TMT—A time	11.21 (11.32)	13.43 (16.21)	
									TMT—A mistakes	0.42 (0.77)	0.25 (0.44)	
									TMT—B time	15.91 (18.68)	15.33 (11.26)	
									TMT—B mistakes	2.14 (1.46)	2.23 (1.52)	
									WAIS-III digits	10.47 (2.61)	10.58 (2.95)	
									WAIS-III digit symbol	9.83 (2.87)	10.11 (2.41)	
									WAIS-III arithmetic	9.75 (3.08)	10.22 (2.82)	
									CAMCOG visual reasoning	2.22 (1.42)	2.43 (1.20)	
									RBMT visual memory	7.94 (2.10)	7.78 (2.38)	
									CDT order	7.35 (2.24)	7.60 (2.38)	
									CDT copy	9.10 (1.14)	8.99 (1.50)	
									Semantic verbal fluency	7.03 (2.88)	7.62 (3.59)	
									Phonological verbal fluency—P	7.56 (3.04)	7.82 (2.76)	
									Phonological verbal fluency—M	7.68 (3.44)	8.26 (3.42)	
									Phonological verbal fluency—R	8.50 (2.57)	8.97 (2.58)	
	26	76.23 (6.56)	9.36 (2.86)	4	Passive Control Training	-	-	-	MMSE	24.15 (4.16)	24.81 (4.06)	
									ADAS-Cog	14.88 (7.75)	14.31 (7.29)	
									TMT—A time	7.80 (5.61)	9.09 (6.66)	
									TMT—A mistakes	0.54 (1.07)	0.64 (1.19)	
									TMT—B time	14.44 (11.02)	15.00 (9.13)	
									TMT—B mistakes	1.59 (1.42)	1.40 (1.31)	
									WAIS-III digits	10.65 (2.17)	11.48 (2.20)	
									WAIS-III digit symbol	10.17 (2.06)	10.35 (2.21)	
									WAIS-III arithmetic	10.50 (2.34)	10.68 (2.67)	
									CAMCOG visual reasoning	2.00 (1.10)	1.96 (1.06)	
									RBMT visual memory	7.00 (3.02)	6.84 (3.01)	
									CDT order	7.25 (2.74)	7.56 (2.07)	
									CDT copy	8.81 (1.86)	8.76 (1.93)	
									Semantic verbal fluency	7.80 (3.28)	7.56 (3.37)	
									Phonological verbal fluency—P	7.72 (3.55)	8.16 (3.22)	
									Phonological verbal fluency—M	7.48 (3.66)	8.28 (3.69)	
									Phonological verbal fluency—R	8.84 (2.53)	9.40 (2.90)	
Djabelkhir et al. [36] (MCI)	9	78.2 (7)	N.R.	4	Computer- based Cognitive Training	12	90	1	MMSE	27 (2)	28 (1.4)	9
									16-FCRT	22.8 (10.7)	25.1 (10)	
									TMT—A	52.1 (18.8)	47 (22.8)	
									TMT—B	135.7 (65.6)	111.5 (65.6)	
									Digit span backward	4 (0.61)	4 (0.61)	
									Phonological verbal fluency	19.6 (9.7)	22.4 (8)	
									Semantic verbal fluency	24.2 (3.8)	25.8 (6)	
	10	75.2 (6.4)	N.R.	3	Active Control Training	12	90	1	MMSE	27.7 (1.9)	27.8 (1.5)	
									16-FCRT	26.6 (8.7)	26.3 (7.5)	
									TMT—A	50.8 (18.3)	41.1 (12.3)	
									TMT—B	112 (19.8)	101.5 (29.2)	
									Digit span backward	4 (0.61)	3.75 (0.82)	
									Phonological verbal fluency	22.1 (6.4)	22.5 (5.9)	
									Semantic verbal fluency	27 (8)	27.4 (7.4)	
Duff et al. [37] (a-MCI)	55	74.9 (6.3)	16.5 (2.8)	29	Computer- based Cognitive Training	12/13	45	4/5	RBANS	85.1 (13.5)	85.6 (14.6)	10
	58	74.9 (5.8)	16 (2.9)	33	Active Control Training	12/13	45	4/5	RBANS	85.7 (14.2)	89.1 (13.5)	
Fiatarone Singh et al. [38] (MCI)	22	N.R.	N.R.	N.R.	Computer- based Cognitive Training	26	75	2/3	ADAS-cog	8.79 (0.98)	7.31 (0.73)	9
									WAIS III similarities	19.11 (1.05)	21.52 (1.08)	
									Matrix reasoning test	11.98 (1.07)	11.82 (1.08)	
									Semantic verbal fluency	20.2 (1.11)	20.5 (1.12)	
									COWAT	38.24 (2.83)	41.85 (2.85)	
									List learning memory	18.59 (0.89)	19.72 (0.90)	
									BVRT	5.98 (0.43)	5.88 (0.44)	
									Logical memory I	10.13 (0.97)	10.61 (0.96)	
									Logical memory delayed recall	8.17 (1.11)	9.08 (1.09)	
									SDMT	45.58 (2.40)	46.8 (2.42)	
	27	N.R.	N.R.	N.R.	Physical and Computer- based Cognitive Training	26	75	2/3	ADAS-cog	8.02 (0.69)	6.26 (0.69)	
									WAIS III similarities	19.05 (1.01)	20.57 (1.01)	
									Matrix reasoning test	12.04 (1.06)	13.26 (1.01)	
									Semantic Verbal Fluency	17.9 (0.55)	18.2 (1.03)	
									COWAT	36.40 (2.66)	37.88 (2.66)	
									List learning memory	20.13 (0.84)	20.79 (0.84)	
									BVRT	5.86 (0.41)	6.27 (0.41)	
									Logical memory I	9.46 (0.90)	10.42 (0.90)	
									Logical memory delayed recall	8.71 (1.03)	8.04 (1.01)	
									SDMT	44.94 (2.25)	47.92 (2.25)	
	24	N.R.	N.R.	N.R.	Active Control Training	26	60	2/3	ADAS-cog	8.09 (0.10)	7.14 (0.70)	
									WAIS III similarities	17.84 (1.01)	19.02 (1.03)	
									Matrix reasoning test	11.53 (1.01)	11.27 (1.03)	
									Semantic verbal fluency	18.4 (1.03)	17.7 (1.05)	
									COWAT	35.23 (2.65)	41.09 (2.69)	
									List learning memory	18.84 (0.83)	19.09 (0.85)	
									BVRT	6.51 (0.41)	5.46 (0.42)	
									Logical memory I	9.60 (0.92)	10.99 (0.90)	
									Logical memory delayed recall	8.17 (1.01)	7.75 (1.03)	
									SDMT	41.68 (2.25)	44.11 (2.29)	
Finn and McDonald [39] (a-MCI)	12	72.83 (5.7)	13.75 (2.8)	8	Computer-based Cognitive Training	4	N.R.	2	VPA immediate recall	5.41 (3.7)	7.75 (4.2)	8
									VPA delayed recall	1.50 (1.3)	2.42 (1.7)	
									TMT—A	49.67 (22.6)	42.67 (15.8)	
									TMT—B	128.92 (47.5)	120.42 (48.2)	
									Symbol span	14.42 (4.4)	16.83 (3.3)	
	12	75.08 (7.5)	13.67 (3.8)	9	Passive Control Training	-	-	-	VPA immediate recall	6.50 (5.8)	8.67 (7.2)	
									VPA delayed recall	2.25 (1.9)	2.08 (1.9)	
									TMT—A	45.42 (12.8)	46.92 (22.7)	
									TMT—B	141.33 (54.4)	115.42 (49.8)	
									Symbol span	14.17 (6)	14.75 (6.2)	
Giuli et al. [40] (MCI)	48	76 (6.3)	6.7 (3.8)	17	Cognitive Training	10	90	1	Digit span forward	4.52 (0.8)	4.68 (0.9)	7
									Digit span backward	2.76 (0.9)	3.04 (0.9)	
									MMSE	25.85 (1.9)	25.62 (2.5)	
									Prose memory	7.05 (3.8)	8.78 (3.9)	
									Word-pairing learning test	8.45 (3.6)	9.6 (4.7)	
									Corsi supraspan	4.84 (0.8)	5.11 (0.8)	
									Semantic verbal fluency	1.87 (1.3)	2 (1.5)	
									Phonological verbal fluency	29.23 (8.2)	30.85 (8.6)	
									Attentive matrices	38.61 (10.1)	42.15 (9.9)	
	49	76.5 (5.7)	5.3 (3)	19	Passive Control Training	-	-	-	Digit span forward	4.69 (0.8)	4.5 (0.8)	
									Digit span backward	2.75 (0.8)	2.40 (0.7)	
									MMSE	25.85 (2.3)	25.43 (3.2)	
									Prose memory	7.2 (4.5)	6.56 (4.1)	
									Word-pairing learning test	6.69 (3.2)	6.41 (2.9)	
									Corsi supraspan	5.04 (0.7)	4.8 (0.9)	
									Semantic verbal fluency	2.19 (1.3)	2.19 (1.3)	
									Phonological verbal fluency	24.39 (7.9)	23.85 (5.9)	
									Attentive matrices	40.75 (9.6)	39.16 (10)	
Greenaway et al. [41] (a-MCI)	20	72.7 (6.9)	16.4 (2.8)	8	Cognitive Training	12	60	2	DRS-2	131.1 (6.3)	131.6 (6.8)	8
									MMSE	26.4 (2.2)	26 (2.9)	
	20	72.3 (7.9)	16.4 (2.8)	7	Passive Control Training	-	-	-	DRS-2	133.8 (4.2)	134.8 (5.1)	
									MMSE	27.2 (2.4)	27.3 (2.2)	
Hagovská and Olekszyová [42] (MCI)	40	68 (4.4)	N.R.	22	Physical and Computer- based Cognitive Training	10	30	2	MMSE	25.97 (2.57)	26.97 (2.21)	8
	38	65.9 (6.2)	N.R.	19	Passive Control Training	-	-	-	MMSE	26.02 (1.47)	26.10 (1.46)	
Herrera et al. [43] (a-MCI)	11	75.09 (1.97)	N.R.	6	Computer- based Cognitive Training	12	60	2	Digit span forward	4.45 (0.31)	4.91 (0.21)	7
									Digit span-backward	3.36 (0.24)	4.00 (0.19)	
									BEM recall test	6.23 (0.35)	7.28 (0.26)	
									16-FCRT	40.55 (0.41)	42.91 (0.76)	
									Rey figure copy	10.09 (1.52)	10.45 (1.36)	
	11	78.18 (1.44)	N.R.	5	Active Control Training	12	60	2	Digit span forward	4.36 (0.24)	4.18 (0.12)	
									Digit span-backward	3.82 (0.18)	3.64 (0.20)	
									BEM recall test	6.40 (0.46)	6.05 (0.25)	
									16-FCRT	41.09 (0.44)	39.91 (0.44)	
									Rey figure copy	11.86 (1.27)	10.23 (0.87)	
Hughes et al. [44] (MCI)	10	78.5 (7.1)	13.8 (2.4)	2	Computer- based Cognitive Training	24	90	1	CAMCI	25.55 (6.24)	29.41 (5.48)	6
	10	76.2 (4.3)	13.1 (1.9)	4	Active Control Training	24	30	1	CAMCI	25.49 (6.34)	25.59 (6.86)	
Hyer et al. [45] (MCI)	34	75.1 (7.4)	N.R.	17	Computer- based Cognitive Training	5–7	40	5	TMT—B	132.38 (47.92)	118.92 (43.49)	9
									Span board	8.79 (2.48)	11.54 (3.37)	
	34	75.2 (7.8)	N.R.	15	Passive Control Training	-	-	-	TMT—B	133.97 (41.56)	112.57 (39.74)	
									Span board	9.73 (3.10)	10.77 (3.07)	
Lam et al. [46] (MCI)	145	74.4 (6.4)	5.2 (4.3)	30	Cognitive Training	16	60	3	MMSE	25.7 (2.4)	25.8 (2.6)	10
									ADAS-Cog	11.3 (3.2)	8.8 (3.5)	
									Word list delayed recall	3.5 (2.2)	5.8 (2.1)	
									Semantic verbal fluency	34.2 (7.3)	36.2 (8.2)	
	132	76.3 (6.6)	5.7 (4.9)	28	Cognitive and Physical Training	16	60	3	MMSE	25.2 (2.3)	25.7 (2.5)	
									ADAS-Cog	11.6 (3.4)	8.9 (3.2)	
									Word list delayed recall	3.2 (2.2)	5.3 (2.1)	
									Semantic verbal fluency	32.8 (6.7)	35.8 (7.2)	
	147	75.5 (6.7)	5.7 (4.3)	34	Active Control Training (Physical)	16	60	3	MMSE	25.8 (2.3)	26.2 (2.2)	
									ADAS-Cog	11.7 (3.3)	8.8 (3.6)	
									Word list delayed recall	2.5 (3.3)	5.7 (2.3)	
									Semantic verbal fluency	33.3 (7.3)	35.7 (8)	
	131	75.4 (6.1)	5.7 (4.9)	29	Active Control Training (Social)	16	60	3	MMSE	25.6 (2.4)	25.8 (2.4)	
									ADAS-Cog	11.5 (3.4)	9.2 (3.3)	
									Word list delayed recall	3.4 (2.1)	5.4 (2.1)	
									Semantic verbal fluency	32.7 (7.4)	34.4 (7.9)	
Law et al. [47] (MCI)	40	74.1 (7.6)	N.R.	17	Computer- based Cognitive Training	10	60	N.R.	CVLT immediate recall	16.35 (0.94)	17.90 (0.61)	7
									CVLT delayed recall	5.26 (0.71)	6.29 (0.29)	
									Semantic verbal fluency	10.43 (0.65)	11.19 (0.46)	
									TMT—A	134.31 (13.76)	123.38 (6.13)	
									TMT—B	228.27 (13.83)	213.38 (8.29)	
	43	73.68 (6.8)	N.R.	16	Active Control Training	10	45/50	N.R.	CVLT immediate recall	15.49 (0.74)	19.84 (0.59)	
									CVLT delayed recall	5.00 (0.51)	7.24 (0.28)	
									Semantic verbal fluency	9.33 (0.56)	12.62 (0.45)	
									TMT—A	136.28 (10.55)	111.51 (5.91)	
									TMT—B	236.97 (13.08)	189.90 (7.99)	
Lin et al. [48] (a-MCI)	10	72.90 (8.23)	N.R.	5	Vision based speed of processing training	6	60	4	UFOV	136.35 (87.42)	63.96 (22.22)	6
	11	73.09 (9.60)	N.R.	6	Active Control Training	6	60	4	UFOV	96.63 (48.67)	87.65 (59.53)	
Mavros et al. [49] (MCI)	24	N.R.	N.R.	N.R.	Cognitive Training	26	N.R.	2/3	ADAS-Cog	8.4 (3.2)	6.7 (3.2)	9
									WAIS III Similarities	8.4 (3.2)	6.7 (3.2)	
									Matrix reasoning test	12.0 (4.6)	12.5 (4.6)	
									Semantic verbal fluency	19.2 (4.4)	19.6 (4.5)	
									COWAT	38.2 (11.3)	40.8 (11.4)	
									BVRT	5.9 (1.7)	6.3 (1.7)	
									Logical memory I	10.8 (3.8)	10.1 (3.8)	
									Logical memory delayed recall	8.7 (4.2)	8.8 (4.2)	
									SDMT	45.3 (9.4)	47.2 (9.5)	
	27	N.R.	N.R.	N.R.	Active Control Training	26	N.R.	2/3	ADAS-Cog	8.2 (3.2)	6.3 (3.3)	
									WAIS III Similarities	8.2 (3.2)	6.3 (3.3)	
									Matrix reasoning test	12.4 (4.6)	13.1 (4.9)	
									Semantic verbal fluency	18.5 (4.5)	19.3 (4.7)	
									COWAT	38.0 (11.3)	43.1 (11.8)	
									BVRT	6.0 (1.7)	5.8 (1.8)	
									Logical memory I	11.7 (3.8)	10.1 (4.1)	
									Logical memory delayed recall	11.7 (4.1)	10.1 (4.4)	
									SDMT	43.1 (9.4)	45.3 (9.7)	
Nousia et al. [50] (MCI)	25	71.20 (5.07)	8.92 (3.37)	6	Computer- based Cognitive Training	15	60	2	WMT immediate recall	19.36 (3.38)	21.00 (2.72)	10
									WMT delayed recall	1.80 (0.76)	3.04 (1.21)	
									Boston naming test	13.56 (1.45)	14.60 (0.65)	
									Semantic verbal fluency	30.44 (7.76)	40.60 (7.17)	
									CDT	13.68 (1.25)	14.44 (0.82)	
									Digit span forward	6.60 (1.32)	6.72 (1.34)	
									Digit span backward	4.48 (1.23)	4.64 (1.08)	
									TMT—A	98.44 (27.31)	80.72 (23.45)	
									TMT—B	222.48 (53.79)	174.16 (37.11)	
	21	71.90 (6.24)	8.42 (3.06)	5	Passive Control Training	-	-	-	WMT immediate recall	19.90 (3.78)	20.57 (2.93)	
									WMT delayed recall	1.43 (1.29)	0.67 (0.58)	
									Boston naming test	13.10 (1.64)	12.90 (2.63)	
									Semantic verbal fluency	38.05 (7.49)	34.90 (5.54)	
									CDT	14.00 (1.34)	13.90 (1.18)	
									Digit span forward	6.33 (1.59)	6.10 (1.58)	
									Digit span backward	4.52 (1.29)	4 (1.30)	
									TMT—A	110.14 (37.02)	113.67 (37.36)	
									TMT—B	238.38 (52.25)	237.86 (43.73)	
Olchik et al. [51] (a-MCI)	16	70.3 (4.3)	14.3 (4.9)	4	Cognitive Training	4	90	2	Semantic verbal fluency	15.2 (4.6)	17.0 (4.1)	7
									Phonological verbal fluency	30.5 (9.8)	35.3 (9.1)	
									RAVLT immediate recall	34.5 (10.3)	40.3 (8.8)	
									RAVLT delayed recall	5.4 (4.0)	7.3 (3.3)	
									RBMT story immediate recall	5.9 (2.0)	8.8 (2.4)	
									RBMT story delayed recall	4.6 (2.6)	8.6 (2.9)	
	14	70.2 (5.7)	11.2 (4.2)	2	Passive Control Training	-	-	-	Semantic verbal fluency	15.4 (3.2)	14.0 (4.4)	
									Phonological verbal fluency	28.9 (10.5)	32.6 (10.4)	
									RAVLT immediate recall	34.5 (8.3)	36.2 (8.8)	
									RAVLT delayed recall	4.2 (2.2)	6.0 (2.6)	
									RBMT story immediate recall	5.0 (2.6)	9.0 (3.2)	
									RBMT story delayed recall	4.7 (3.1)	7.3 (3.1)	
Poptsi et al. [52] (a-MCI)	150	68.12 (6.31)	11.31 (4.19)	31	Physical and Computer- based Cognitive Training	N.R.	N.R.	N.R.	MMSE	27.70 (1.82)	27.90 (2.65)	10
									RAVLT immediate recall	10.55 (2.59)	11.78 (2.47)	
									RAVLT delayed recall	7.68 (3.23)	9.65 (3.29)	
									RBMT history recall	11.42 (3.90)	11.13 (3.62)	
									Rey figure copy	12.94 (6.34)	15.60 (7.06)	
									RBMT working memory	12.39 (3.53)	11.85 (3.49)	
									Phonological verbal fluency	9.77 (3.47)	12.00 (3.34)	
									TEA	47.00 (11.72)	47.29 (11.72)	
	133	67.11 (9.10)	10.69 (4.68)	22	Passive Control Training	N.R.	N.R.	N.R.	MMSE	27.28 (2.24)	26.79 (3.47)	
									RAVLT immediate recall	10.73 (4.15)	10.75 (3.97)	
									RAVLT delayed recall	7.90 (3.25)	7.80 (3.77)	
									RBMT history recall	11.06 (3.67)	9.82 (4.33)	
									Rey figure copy	11.71 (6.42)	12.98 (6.25)	
									RBMT working memory	12.11 (3.39)	10.72 (3.90)	
									Phonological verbal fluency	10.26 (4.15)	10.05 (3.69)	
									TEA	47.82 (12.63)	43.83 (14.82)	
Rapp et al. [53] (MCI)	9	73.33 (6.61)	N.R.	1	Cognitive Training	6	120	1	Word list immediate recall	8.11 (3.02)	11.56 (2.83)	8
									Word list delayed recall	3.56 (2.92)	8.44 (4.22)	
	10	75.10 (7.03)	N.R.	7	Passive Control Training	-	-	-	Word list immediate recall	5.10 (0.99)	7.80 (3.22)	
									Word list delayed recall	1.90 (1.45)	4.70 (3.62)	
Rojas et al. [54] (MCI)	15	72 (14.29)	10.53 (3.78)	9	Cognitive Training	24	120	1	MMSE	27.53 (2.33)	27.53 (2.00)	9
									Memory free recall	11.07 (1.33)	10.64 (1.74)	
									Boston naming test	44.20 (7.25)	47.07 (9.20)	
									Semantic verbal fluency	13.47 (3.09)	16.50 (3.67)	
									Phonological verbal fluency	10.47 (4.64)	11.93 (4.46)	
	15	77.93 (7.5)	10.53 (3.85)	8	Passive Control Training	-	-	-	MMSE	27.13 (2.10)	25.36 (2.53)	
									Memory free recall	9.64 (2.22)	8.64 (2.34)	
									Boston naming test	42.93 (6.78)	43.14 (8.10)	
									Semantic verbal fluency	13.47 (3.66)	11.07 (3.40)	
									Phonological verbal fluency	10.50 (3.91)	9.07 (3.91)	
Schmitter-Edgecombe and Dyck [55] (MCI)	23	72.96 (7.05)	14.48 (2.81)	7	Cognitive Training	10	120	2	RBMT-II	15.35 (5.31)	17.35 (6.11)	8
									RBANS immediate memory	84.91 (17.37)	92.78 (19.85)	
									RBANS delayed memory	79.39 (19.59)	86.10 (24.50)	
	23	73.35 (7.89)	15.78 (3.32)	12	Passive Control Training	-	-	-	RBMT-II	15.22 (6.99)	15.13 (6.85)	
									RBANS immediate memory	87.56 (18.64)	88.52 (23.15)	
									RBANS delayed memory	77.57 (23.53)	78.52 (26.25)	
Savulich et al. [56] (a-MCI)	21	75.2 (7.4)	15.9 (1.3)	11	Computer- based Cognitive Training	4	60	2	MMSE	26.6 (2.9)	27.4 (1.5)	9
	21	76.9 (8.3)	16 (2.1)	14	Passive Control Training	-	-	-	MMSE	26.8 (2.2)	26.1 (2.4)	
Sukontapol et al. [57] (MCI)	30	N.R.	N.R.	7	Cognitive Training	N.R.	180	N.R.	MoCA	21.37 (2.04)	25.40 (1.58)	8
	30	N.R.	N.R.	14	Passive Control Training	-	-	-	MoCA	18.43 (4.06)	18.77 (4.35)	
Tsolaki et al. [58] (MCI)	122	68.45 (6.99)	9.31 (4.11)	32	Cognitive Training	N.R.	90	3	MMSE	28.09 (1.59)	29.00 (6.18)	9
									MoCA	22.98 (3.36)	24.71 (3.05)	
									FUCAS planning	6.20 (0.60)	6.04 (0.25)	
									Rey figure copy	29.85 (5.89)	31.53 (5.30)	
	79	66.86 (8.79)	8.97 (4.19)	18	Passive Control Training	-	-	-	MMSE	27.59 (1.88)	27.06 (2.34)	
									MoCA	22.20 (3.54)	22.45 (4.78)	
									FUCAS planning	6.18 (0.56)	6.18 (0.56)	
									Rey figure copy	28.48 (8.24)	29.18 (7.19)	
Weng et al. [59] (MCI)	33	81.82 (11.28)	N.R.	4	Computer- based Cognitive Training	8	40–60	2	MoCA	17.45 (4.65)	18.09 (4.71)	9
									Digit span forward	4.42 (1.25)	4.55 (1.23)	
									Digit span backward	2.45 (0.94)	2.73 (0.91)	
									WAIS-IV digit symbol	19.03 (8.12)	21.48 (6.70)	
									Phonological verbal fluency	8.94 (3.03)	9.06 (2.68)	
									WAIS-IV similarities	10.61 (4.96)	11.06 (4.44)	
	29	80.72 (9.91)	N.R.	1	Active Control Training	8	40–60	2	MoCA	18.41 (3.40)	17.86 (3.32)	
									Digit span forward	4.03 (1.12)	3.76 (1.12)	
									Digit span backward	2.69 (0.97)	2.52 (0.91)	
									WAIS-IV digit symbol	21.45 (7.78)	20.10 (8.03)	
									Phonological verbal fluency	9.59 (2.71)	9.66 (2.76)	
									WAIS-IV similarities	11.17 (3.96)	11.55 (4.37)	

Values are shown as mean (SD). Abbreviations: N.R. = not reported; n M = number of male participants; N = sample size; SD = standard deviation; MCI = mild cognitive impairment; a-MCI = anamnestic mild cognitive impairment; ADAS-Cog = Alzheimer’s disease assessment scale-cognitive subscale; BEM = 12 word-list recall test from the BEM-144 memory battery; BVRT = Benton visual retention test; CAMCOG = Cambridge cognition examination; CDT = clock drawing test; COWAT = controlled oral word association test; FUCAS = functional cognitive assessment; MMSE = mini-mental state examination; MoCA = Montreal cognitive assessment; RAVLT = Rey auditory verbal learning test; RBANS = repeatable battery for the assessment of neuropsychological status; RBMT = Rivermead behavioural memory test; SDMT = symbol digit modalities test; TEA = visual selective attention; TMT = trail making test; UFOV = useful field of view; WAIS = Wechsler adult intelligence scale; VPA = verbal paired associates test; DRS-2 = dementia rating scale-2; CAMCI = computerized assessment of mild cognitive impairment; CVLT = California verbal learning test; FCRT = free and cued reminding test; WMT = word memory test.

**Table 2:** Summary of meta-analytic results of the following cognitive domains including studies comparing patients with MCI who underwent cognitive training and those without cognitive training.

Domain/Outcomes	K	N	EG	CG	Pooled Effect Size Hedges’ *g* (*p* Value)	(95% Confidence Intervals)		Homogeneity Statistics			Egger’s *t* Test for Publication Bias (*p* Value)	Trim and Fill (Estimated Effect Size)
						LL	UL	Q (df)	P	I^2^		
**Global Cognitive Function** *(Mini Mental State Examination; 66.6%)*	12	1543	799	744	0.21 **(0.001)**	0.08	0.33	13.88 (11)	0.240	20.73	1.61 (0.139)	2 [0.18 (0.005)]
**Memory**												
- **Short-Term Verbal Memory** *(Digit Span-Forward; 100%)*	8	398	216	182	0.79 **(0.029)**	0.08	1.50	73.09 (7)	<0.001	90.42	0.87 (0.417)	0
- **Long-Term Verbal Memory** *(Rey Auditory Verbal Learning Test-Delayed Recall; 22.2%)*	4	449	227	222	0.31 **(0.044)**	0.01	0.62	5.65 (3)	0.130	46.86	−4.72 (0.042)	0
*Sensitivity Analysis after removing Ciarmiello et al., 2015* [33]	3	419	212	207	0.39 **(0.014)**	0.08	0.69	3.69 (2)	0.158	45.80	−2.64 (0.231)	0
**Executive Functions**												
- **Shifting** *(Trail Making Test—Part B; 100%)*	6	302	156	146	−0.60 (0.339)	−1.83	0.63	115.42 (5)	<0.001	95.67	−0.69 (0.529)	2 [−1.18 (0.056)]
- **Abstraction ability/Concept Formation** *(Matrix Reasoning Test; 57.1%)*	4	205	125	80	0.41 (0.069)	−0.03	0.84	6.71 (3)	0.082	55.32	−0.52 (0.652)	0
- **Generativity** *(Phonological Verbal Fluency; 80%)*	8	653	344	309	0.77 **(0.037)**	0.05	1.49	111.67 (7)	<0.001	93.73	0.50 (0.635)	0
- **Working Memory** *(Digit Span-Backward; 70%)*	7	317	173	144	0.78 **(0.003)**	0.27	1.29	25.07 (6)	<0.001	76.06	1.09 (0.325)	0
**Processing Speed/Attention** *(Trail Making Test—Part A; 50%)*	5	234	122	112	−0.31 (0.594)	−1.43	0.82	63.97 (4)	<0.001	93.75	0.43 (0.697)	1 [−0.56 (0.284)]
**Visuospatial and Constructional Ability** *(Rey Osterrieth Complex Figure-Copy Task; 66.6%)*	4	536	298	238	0.49 **(0.022)**	0.07	0.91	11.39 (3)	0.010	73.67	4.13 (0.054)	1 [0.31 (0.215)]
**Language** *(Semantic Verbal Fluency; 100%)*	11	1116	576	540	−0.08 (0.828)	−0.75	0.60	216.78 (10)	<0.001	95.39	−0.25 (0.810)	4 [−0.85 (0.032)]

In brackets are reported the names of the tests used as the basis for computing the effect sizes and the percentage of studies that utilized these tests for each cognitive domain considered as the outcome of the meta-analysis. Abbreviations: K = number of studies; N = total number of participants; EG = experimental group; CG = control group; LL = lower limit; UL = upper limit; Q and I^2^ = heterogeneity statistics; df = degrees of freedom; statistically significant values are presented in bold.

## Data Availability

The data presented in this study are available in the article and in the Supplementary Material.

## Supplementary Materials

The following supporting information can be downloaded at: https://www.mdpi.com/article/10.3390/brainsci13111510/s1.
