# Supplementary material for: The Efficacy of Cognitive Training on Neuropsychological Outcomes in Mild Cognitive Impairment: A Meta-Analysis
Source: Brain Sci. 2023 Oct 25;13(11):1510. doi: 10.3390/brainsci13111510 (PMC10669748; doi:10.3390/brainsci13111510)
Supplement: Supplementary file 1 [file brainsci-13-01510-s001.zip › brainsci-2633297-supplementary.pdf]

Supplementary Material S1. Classification of neuropsychological tasks for each cognitive domain.

COGNITIVE DOMAINS	TEST	REFERENCE
**Global Cognitive Functioning**	**Mini Mental State Examination**	**Barban et al., 2016; Ciarmiello et al., 2015; Diaz Baquero et al., 2022; Djabelkhir et al., 2017; Giuli et al., 2016; Greenaway et al., 2013; Hagovská and Olekszyová, 2016; Lam et al., 2015; Poptsi et al., 2022; Rojas et al., 2013; Savulich et al., 2017; Tsolaki et al., 2011**
	Alzheimer's Disease Assessment Scale: Cognitive Subscale	Diaz Baquero et al., 2022; Fiatarone Singh et al., 2014: Lam et al., 2015; Mavros et al., 2017
	Montreal Cognitive Assessment	Sukontapol et al., 2018; Tsolaki et al., 2011; Weng et al., 2019
	Computerized Assessment of Mild Cognitive Impairment	Hughes et al., 2014
	Dementia Rating Scale- 2	Greenaway et al., 2013
	Repeatable Battery for the Assessment of Neuropsychological Status	Duff et al., 2022
**Memory**		
- Short Term:		
Verbal	**Digit Span-Forward**	**Balietti et al., 2016; Carretti et al., 2013; Ciarmiello et al., 2015; Combourieu-Donnezan et al., 2018; Giuli et al., 2016; Herrera et al., 2012; Nousia et al., 2021; Weng et al., 2019**

	Verbal Span	Ciarmiello et al., 2015
Visuospatial	Corsi's Test	Ciarmiello et al., 2015
- Long Term:		
Verbal	California Verbal Learning Test-II: Immediate	Law et al., 2014
	California Verbal Learning Test-II: Delayed Recall	Law et al., 2014
	Rey Auditory Verbal Learning Test-Immediate Recall	Ciarmiello et al., 2015; Poptsi et al., 2022; Olchik et al.; 2013
	**Rey Auditory Verbal Learning Test-Delayed Recall**	**Barban et al., 2016; Ciarmiello et al., 2015; Poptsi et al., 2022; Olchik et al.; 2013**
	Logical Memory-Immediate Recall	Fiatarone Singh et al., 2014; Mavros et al., 2017
	Logical Memory-Delayed Recall	Fiatarone Singh et al., 2014; Mavros et al., 2017
	List Learning Memory	Fiatarone Singh et al., 2014
	Story Immediate Recall	Olchik et al.; 2013
	Story Delayed Recall	Olchik et al.; 2013; Poptsi et al., 2022
	Prose Memory	Balietti et al., 2016; Ciarmiello et al., 2015; Giuli et al., 2016
	Word Pairing Test	Balietti et al., 2016
	12-Word-List Recall Test	Herrera et al., 2012
	16- Free and Cued Reminding Test	Djabelkhir et al., 2017; Herrera et al., 2012

	Verbal Paired Associates Immediate Recall	Finn and McDonald, 2015
	Verbal Paired Associates Immediate Recall	Finn and McDonald, 2015
	Word Memory Test Immediate Recall	Nousia et al., 2021
	Word Memory Test Delayed Recall	Nousia et al., 2021
	Memory Free Recall	Rojas et al., 2013
	Rivermead Behavioral Memory Test	Schmitter-Edgecombe and Dyck, 2014
	Repeatable Battery for the Assessment of Neuropsychological Status- Immediate Recall	Schmitter-Edgecombe and Dyck, 2014
	Repeatable Battery for the Assessment of Neuropsychological Status- Delayed Recall	Schmitter-Edgecombe and Dyck, 2014
	Word list-Immediate Recall	Rapp et al., 2002
	Word list-Delayed Recall	Lam et al., 2015; Rapp et al., 2002
	List Recall	Carretti et al., 2013
	Word Pairing Learning Test	Giuli et al., 2016
Spatial	Rey Osterrieth Complex Figure- Delayed recall	Ciarmiello et al., 2015
	Corsi Supraspan	Balietti et al., 2016; Giuli et al., 2016
	Benton Visual Retention Test	Fiatarone Singh et al., 2014; Mavros et al., 2017
	Visual Memory	Diaz Baquero et al., 2022

**Executive Functions**		
- Set-shifting	**Trail Making Test: Part B**	**Diaz Baquero et al., 2022; Djabelkhir et al., 2017; Finn and McDonald, 2015; Hyer et al., 2016; Law et al., 2014; Nousia et al., 2021**
- Abstraction Ability/Concept Formation	Similarities	Fiatarone Singh et al., 2014; Mavros et al., 2017; Weng et al., 2019
	**Matrix Reasoning Test**	**Ciarmiello et al., 2015; Combourieu-Donnezan et al., 2018; Fiatarone Singh et al., 2014; Mavros et al., 2017**
	Visual Reasoning	Diaz Baquero et al., 2022
	Cattel Test	Carretti et al., 2013
- Inhibition	Stroop Test-Interference	Combourieu-Donnezan et al., 2018
- Generativity	**Phonological Verbal Fluency**	**Balietti et al., 2016; Diaz Baquero et al., 2022; Djabelkhir et al., 2017; Giuli et al., 2016; Olchik et al.; 2013; Rojas et al., 2013; Poptsi et al., 2022; Weng et al., 2019**
	Controlled Oral Word Association Test	Fiatarone Singh et al., 2014; Mavros et al., 2017
- Pianification	Planning	Tsolaki et al., 2011
- Working Memory	**Digit Span-Backward**	**Carretti et al., 2013; Combourieu-Donnezan et al., 2018; Djabelkhir et al., 2017; Giuli et al., 2016; Herrera et al., 2012; Nousia et al., 2021; Weng et al., 2019**
	Working Memory	Poptsi et al., 2022
	Symbol Span	Finn and McDonald, 2015
	Span Board	Hyer et al., 2016
	Dot Matrix	Carretti et al., 2013

**Processing Speed/Attention**	**Trail Making Test: Part A**	**Diaz Baquero et al., 2022; Djabelkhir et al., 2017; Finn and McDonald, 2015; Law et al., 2014; Nousia et al., 2021**
	Digit Symbol Substitution Test	Diaz Baquero et al., 2022
	Attentional Matrices	Balietti et al., 2016; Giuli et al., 2016
	Digit Symbol	Diaz Baquero et al., 2022; Weng et al., 2019
	Visual Selective Attention (TEA)	Poptsi et al., 2022
	Arithmetic	Diaz Baquero et al., 2022
	Pattern comparison task	Carretti et al., 2013
**Visuospatial/Constructional Ability**	**Rey Osterrieth Complex Figure-Copy task**	**Ciarmiello et al., 2015; Herrera et al., 2012; Poptsi et al., 2022; Tsolaki et al., 2011**
	Clock Drawing Test	Diaz Baquero et al., 2022; Nousia et al., 2021
**Language**	**Semantic Verbal Fluency**	**Balietti et al., 2016; Diaz Baquero et al., 2022; Djabelkhir et al., 2017; Fiatarone Singh et al., 2014; Giuli et al., 2016; Lam et al., 2015; Law et al., 2014; Mavros et al., 2017; Nousia et al., 2021; Rojas et al., 2013; Olchik et al., 2013**
	Boston Naming Test	Nousia et al., 2021; Rojas et al., 2013

In bold are reported the neuropsychological tasks inserted as outcomes in the meta-analysis.
